# The use of international comparison as interactive teaching method in pharmacy education

**DOI:** 10.1371/journal.pone.0338269

**Published:** 2025-12-11

**Authors:** Jawza Alsabhan, Hailah Almoghirah, Noha Alaloola

**Affiliations:** Department of Clinical Pharmacy, College of Pharmacy, King Saud University, Riyadh, Saudi Arabia; Jouf University, SAUDI ARABIA

## Abstract

**Background:**

Learning through international comparison assignments can significantly impact students’ academic performance.

**Materials and methods:**

This cross-sectional study used a mixed methods approach that included quantitative and qualitative data collection. The survey was divided into five domains: knowledge development, international perspective, future prospects, personal enjoyment, and skills. Students’ levels of agreement were gauged using a five-point Likert scale, with five indicating the strongest agreement and one indicating the strongest disagreement. Additionally, open-ended questions encouraging students to reflect about their experiences were included in the survey.

**Results:**

A total of 214 students completed the questionnaire, achieving a response rate of 81.6%. Nearly 90% of the participants agreed or strongly agreed that a comparison of the two international perspectives was effective in supporting their knowledge of drug approval processes in Saudi Arabia and other countries. However, approximately three-quarters of the students strongly agreed or agreed that international comparison and contrast assignments would influence their career choices. The difference between male and female participants was statistically significant in the International Perspective domain and Skills domain (p = 0.02 and p = 0.018 respectively). The students were enthusiastic about the assignment’s development and improvement of a set of crucial abilities, including searching, analyzing, summarizing, critical thinking, and teamwork, which are crucial for healthcare students, particularly pharmacy students.

**Conclusion:**

Overall, learning by using an international comparison of contrast assignments as an interactive teaching method can positively impact students’ academic and professional development by helping them develop critical thinking, research, writing, cultural awareness, and communication skills.

## Background

Pharmacy students need to be taught regulation and ethics in their pharmacy curricula and programs for several reasons. The most important reason for this is that pharmacists have the responsibility to ensure that they provide the best possible care to their patients as healthcare professionals. This includes ensuring that they adhere to ethical standards and legal requirements [1]. In addition, pharmacists are responsible for dispensing medications, which can have a significant impact on patient safety. Consequently, pharmacists can make decisions that prioritize patient safety by having a solid understanding of ethical standards and regulatory obligations [2]. Moreover, pharmacists must be aware of several laws and rules, including those relating to drugs, privacy, and licensing and ensure compliance with them [3]. Learning regulation and ethics is an essential part of pharmacy students professional development. It helps them develop critical thinking skills, ethical decision-making abilities, and an understanding of the broader context in which they practice in pharmacies [2–5].

Assisting students in developing and understanding pharmacy laws and regulations related to patient health, medication use, and pharmacists’ responsibilities can be addressed by implementing a pharmacy regulation and ethics course in pharmacy colleges. Our pharmacy regulation and ethics course introduce students to pharmacy laws, ethics, and regulations in the pharmacy profession. The main objectives of this course are to: understand the structure of pharmacy law; enhance students’ knowledge regarding the meaning of ethical dilemmas and their reasons; understand the pharmacy codes of ethics and their principles; highlight different ethical issues faced by pharmacists in different areas of practice and research; enable students to evaluate and solve ethical dilemmas using ethical principles and theories; recognize the governmental regulations of drug approvals, drug companies and their products registrations, drug manufacturing, medication pricing, health advertisements, and health education; improve students’ ability to display respect for patients, paying particular attention to attitude, behavior, health beliefs, lifestyle, as well as cultural, ethnic, and socioeconomic factors that influences patient care; understand the legal and ethical implications of intervention during life threatening situations such as poisoning or drug overdose; enhance students’ ability to place health care and professional issues within appropriate historical, cultural, social, economic, scientific, political, and philosophical frameworks; and understand the governmental regulations for narcotics and psychiatric medications.

To deliver legal and ethical considerations appropriately to pharmacy students, there is a need to design an educational strategy using the interactive teaching technique that initially emphasizes the needs of students and gives them the chance to actively engage in the learning process [6]. Interactive teaching plays a crucial role in pharmacy education, as it helps students actively engage in the learning process and develop critical thinking skills [7]. It involves the use of various teaching methods such as group discussions, case studies, role-playing, simulations, and hands-on activities that encourage the students to actively participate in the learning process [8]. By giving students a more interesting and fulfilling learning experience, interactive teaching is often a successful strategy to raise the standard of pharmacy education [6]. Project-based learning (PBL) is a teaching method that involves students working on a project over an extended period to investigate and respond to complex questions, problems, and challenges [9]. Applying the four stages of PBL, planning, research, production, and presentation, allows students to develop critical thinking skills, collaborate with others, and apply what they learned in real-world situations. It also helps students develop communication skills as they present their projects to others [10]. Global perspectives are important in teaching because they help students understand and appreciate different cultures and perspectives [11]. This can lead to increased empathy and understanding of others as well as better problem-solving skills, as the students learn to consider multiple viewpoints. In addition, international practice experience empowers students by providing them with the knowledge, skills, and attitudes required to meet the needs of the current and future generations at a local and global level; this will ensure the accountability of institutions, professional organizations, and political systems to provide job opportunities for future graduates and address the training of support personnel who can work in different settings [12]. The globalization of pharmacy education is a worthy attempt for academics and other stakeholders [13].

Comparative assignments are useful in teaching because they allow students to compare different ideas, cultures, or perspectives [14]. This can help them develop critical thinking skills by analyzing the similarities and differences between different concepts or ideas. It also helps them understand how different cultures approach similar problems and challenges [15]. Although there is a growing amount of research on the topic of internationalizing pharmacy school curricula, there is a lack of data that reflects what students believe and are aware of regarding learning about other cultures, particularly in the context of classroom-based methodologies [12,16].

The major objective of this study is to identify students’ perceptions of using the international comparison as an interactive teaching method in learning ethical regulation and law courses. This may be useful for bringing about awareness in other colleges and schools of pharmacy that may be considering the establishment of an international exchange program for their students.

## Materials and methods

### Study design and participants

This cross-sectional study used an online survey methodology targeting all the students enrolled in a pharmacy regulations and ethics course (PHCL437). The Pharmacy Regulations and Ethics course introduces the concept and application of Saudi regulations and laws to pharmacy practice, making it a crucial course within the Pharm D degree program (fourth year). In this course, the students must undertake group projects as assignments. One such project is a comparison of drug approval-related policies between Saudi Arabia (SA) and other countries. This compare-contrast method is used to improve learners’ cognitive abilities in terms of creativity, analysis, and assessment. Based on the modification of Bloom’s taxonomy, these three cognitive processes are recognized as the most significant objectives of learning and education [17].

The recruitment period started on October 12, 2022, and ended on February 3, 2023. Each student in the class was assigned to a group (each group consisted of 3 or 4 students), and each group collaborated to compare the drug approval process in SA with that in one assigned country. These countries included the USA, Germany, the UK, Australia, China, Japan, Jordan, Brazil, Malaysia, and Canada. The assignments were required to be submitted as a written reflection, not exceeding 750 words, and had to be concise, clear, and comprehensive. The comparison needed to introduce the Saudi drug approval process, describe the drug approval process in the assigned country, and discuss similarities and differences in the drug approval process by providing references. This was done to encourage the students to engage in independent research and inquiry.

The assignment description, objectives, grading criteria, and process of writing an academic comparison and contrast essay were explained to the students before the beginning of the course and were asked to complete the consent form to participate as research participants. The essay assessment guide with grading criteria was made accessible to the students through the course syllabus. This task accounted for 12% of the students’ final grade in the course.

### Questionnaire design

An online survey was conducted to evaluate the students’ impressions of their learning from the comparison assignment The survey development was based on a literature review and modifications of a previous research instrument [7,16]. The survey consisted of 12 questions arranged into five domains of inquiry: knowledge development, international perspective, future prospects, personal enjoyment, and Skills. A five-point Likert scale ranging from 5 (strongly agree) to 1 (strongly disagree) was used to measure the students’ level of agreement. The survey also included five open-ended questions that invited the students to write about their experiences. (A copy of the questionnaire is available at the following link https://forms.gle/4y64aQ61DK6V4RxF8 and in S1 Appendix). A group of researchers reviewed the survey content, clarity, and items related to the study objectives (i.e., face and content validity). The survey was then pilot-tested by conducting cognitive interviews with a purposive sample of students (N = 6). The recommendations from the pilot participants were managed. The responses from these individuals were not included in the final analysis.

### Data analysis

Quantitative data were analyzed using the Statistical Package for the Social Sciences (SPSS). Descriptive statistics, such as frequencies and percentages, were used to summarize the survey responses. Qualitative data were analyzed using Thematic analyzes to analyze the written reflection. A deductive approach where used.

### Sample size

A sample size of 262 was calculated based on the number of students in the College of Pharmacy at King Saud University (816 students) with an estimation of a margin error of 0.05, and a confidence level of 0.95.

### Ethical considerations

A written consent was obtained from all participants before data collection. The students’ participation was completely voluntary, and their decision to participate or not will not affect their current or future relationships with the researchers, course teaching staff, the College of Pharmacy, or King Saud University. This was clarified to the students in the Participant Information Statement (PIS), which was sent to them along with the invitations through a university learning management system (LMS).

A faculty member with academic expertise, who was not involved in the teaching or evaluation of the course, sent out invitations and reminders, both in class and via the course page on the LMS.

This research was reviewed and approved by King Saud University Medical City KSUMC Ethics Review Committee at King Saud University (Approval: E-22-7291).

## Results

Two-hundred and fourteen student’s responses were received from 96 males (44.9%) and 118 female students (55.1%). As all registered students were invited to participate and the majority responded, out of the 262 students registered in the course, 214 completed the survey, yielding a response rate of 81.6%.

### Students’ perceptions

Approximately 90% of the respondents agreed or strongly agreed that a comparison of two international perspectives was effective in supporting their knowledge of drug approval processes in SA and other countries. Most of the students (89%) agreed that the comparison assignments on pharmacy regulations helped them develop international perspectives.

### Perceived impact on learning and understanding

Regarding the impact of learning through international comparison and contrast assignments, approximately three-quarters of the students (70%) strongly agreed or agreed that international comparison and contrast assignments would impact their career choice. Most of the students (86%) agreed or strongly agreed that contrast learning approaches were entertaining and enjoyable and 87.4% of them agreed that learning through international comparisons and contrast assignments could have a significant impact on their research skills. The results are summarized in Table 1.

**Table 1:** Pharmacy student’s response to five domains international comparison survey.

	Strongly Agree/ Agree (%)	Neither agree nor disagree (%)	Strongly disagree/ Disagree (%)	Mean (SD)
**Knowledge development**				
Learning through the international compare/contrast assignment was effective in helping me to understand the drug approval process in Saudi Arabia and the other countries.	**91.1**	**7.9**	**1**	**2.9** **(0.3)**
Learning through the international compare/contrast assignment broadened my awareness of how my profession fits into complex regulatory frameworks.	**92.5**	**7**	**0.5**	**2.9** **(0.2)**
**International perspective**				
Learning through the international compare/contrast assignment was effective in helping me develop an appreciation of how other international societies and cultures regulate the drug approval process	**89.7**	**8.4**	**1.9**	**2.8** **(0.3)**
**Future prospective**				
Learning through the international compare/contrast assignment is relevant to my future aspirations.	**71.5**	**20.6**	**7.9**	**2.6** **(0.6)**
Learning through the international compare/contrast assignment has raised my interest in working abroad one day.	**70.1**	**21**	**8.9**	**2.6** **(0.6)**
**Personal like/ enjoyment**				
Learning through the international compare/contrast assignment was interesting to me and kept me engaged with the material.	**87**	**15**	**7**	**2.6** **(0.6)**
Learning through the international compare/contrast assignment resulted in me learning things that were unexpected or even surprising.	**75.3**	**21**	**3.7**	**2.7** **(0.5)**
**Skills**				
Learning through the international compare/contrast assignment improve my information searching skills.	**87.4**	**8.4**	**4.2**	**2.7** **(0.5)**
Constructing an international compare/contrast assignment developed my ability to analyze and critically evaluate information and evidence.	**85.5**	**11.2**	**3.3**	**2.7** **(0.5)**
Constructing an international compare/contrast assignment developed my ability to generate awareness and ideas appropriate to my profession.	**88.8**	**7.9**	**3.3**	**2.8** **(0.4)**
Learning through international compare/contrast assignment improve my communication and team work skills.	**82.7**	**11.2**	**6.1**	**2.8** **(0.4)**
Constructing an international compare/contrast assignment developed my ability to Innovate	**79**	**15**	**6.1**	**2.8** **(0.4)**

**Fig 1:**
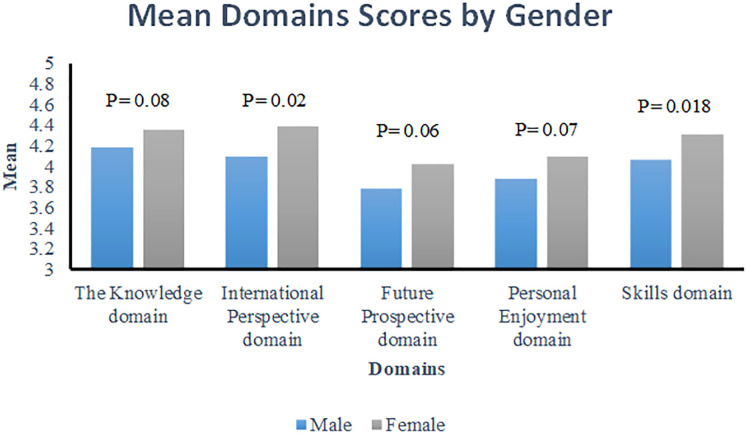
Mean domains scores by gender.

A comparison between male and female participants was conducted to examine differences in mean scores across the various domains and presented in Fig 1. The difference was not statistically significant in the Knowledge domain (p = 0.08), in the Future Prospective domain (p = 0.06) and in the Personal Enjoyment domain, (p = 0.07) while the difference in International Perspective domain and Skills domain, were statistically significant (p = 0.02 and p = 0.018 respectively).

### Reflections on the comparison of two international perspectives

Comments and responses to the open-ended questions were obtained from 214 students. Sample of Student Comments in S2 Appendix. The open-ended questions concerned five aspects: knowledge development, international perspective, future prospects, personal like/enjoyment, and assessment skills.

**In the knowledge development section,** the students’ comments confirmed that the international comparison assignment improved their knowledge of the regulatory processes and drug approval procedures in SA, and their differences and similarities with other countries. Because each group was assigned a different country, a few comments suggested that every group should present their work to the other groups to expand their knowledge of the other countries.

“*It’s interesting how each country has its own method, which makes me more fascinated by learning about the regulations of each country*” (S23).

“*I really learned a lot doing research for the assignment, especially as a pharmacist who’s interested in the field of approval processes. But I think that we can learn even more and better utilize the information that we already have to make a presentation as groups (about the international process alone) to get to know other countries as well*” (S72).

**In the international perspective section,** the students indicated that this assignment helped them become aware that every country has its own regulations and procedures, and despite some similarities, there are differences due to many factors such as culture, geography, and religion.

“*The drug approval process differs from one place to another, despite having the same core values. In Saudi Arabia, and many other Arab and Muslim countries, we strongly consider a religious aspect to the drug approval process. Cultural and geographical differences will set one country apart from the others*” (S148).

**In the future prospective section,** the students indicated that the assignment helped them learn more about a pharmacist’s role in the Saudi Food and Drug Authority (SFDA) in SA and other regulatory agencies in other countries. Thus, it helped them acquire background knowledge for a future career, regardless of whether they choose to work in SA or abroad.

“*It is giving a preconception about the role of pharmacist in drug regulation process and what is the real responsibilities that I will deal with if I become part of the process*” (S11).

“*It’s going to have an impact on my choices of working abroad as the regulations are different for each country*” (S118).

However, some students indicated that this was not their field of interest; therefore, this assignment may not have an impact on their future perceptions.

“*My interest in my future profession comes first from direct contact with the patients or students in my country, and by saying that, I don’t think that regulatory affairs or working abroad would be in my interest from my present or future perspective. At least, that is my opinion at this moment after this assignment*” (S95).

**In the assessment skills section,** the students were excited about how the assignment managed to develop and refine a set of essential skills, such as searching, analyzing, summarizing, critical thinking, and teamwork.

“*This assignment was another necessary step in our progress to graduate with excellent research and critical thinking skills*” (S148).

“*This assignment requires several skills to have or even to take the chance and develop it, and it includes writing skills, communication, cooperation skills, respect for other perspectives and opinions, unity in determining the final document to be submitted, and time management*” (S95).

## Discussion

This study examined pharmacy students’ perceptions of the International Drug Regulation Comparison Project, which is an interactive teaching technique used in pharmacy regulation and ethics courses. This technique has gained student acceptance. Pharmacy students showed a positive perception of the impact of international comparison assignments on several aspects, including knowledge, personal enjoyment, future prospects, and skills. Overall, the findings indicate that gender had a significant effect on the International Perspective and Skills domains, with females scoring higher than males. However, no significant gender differences were observed in the Knowledge, Future Prospective, or Personal Enjoyment domains.

Key knowledge, understanding, and skills are the three basic elements of PBL that focus on students’ learning goals. These goals include standard-based content and skills such as critical thinking, problem-solving, collaboration, and self-management. This study showed that implementing an international drug regulation comparison project in pharmacy regulation and ethics courses helps pharmacy students obtain knowledge and skills in different areas. The students in this study indicated they could recognize cultural differences and similarities by comparing themselves with other cultures and countries. This helped improve their cultural awareness. Moreover, they indicated that the comparison assignment helped them obtain knowledge about drug approval policies and procedures in SA and other countries.

Creating international opportunities in the classroom helped the students expand their perspectives on pharmacy practice and develop their professional vision [14,16,18]. The students in this study perceived that the international comparison assisted them in identifying their career pathway because the assignment helped them learn more about the pharmacist’s role in regulatory agencies in SA and other countries. These findings are consistent with those of a previous study that assessed Australian pharmacy students’ perceptions of taking pharmacy law and practice courses through an international comparison. Researchers have indicated that students perceive that a comparison assignment improves their knowledge and skills and influences their career prospects [16].

Students’ abilities to think critically and practice good quality writing are important skills that they need in their education. The literature shows that using comparisons in teaching and assessments improves student skills [19]. Students in this study perceived that their ability to think critically improved as they had to assess materials from different resources to complete their assignments. Additionally, they perceived that this assignment affected their writing skills, as most of the students indicated that their writing abilities, including the rational structuring of thoughts, utilizing proper language and tone, and appropriately referencing sources, were developed by this comparison project.

This study limitation was assessing the students’ perceptions only, and not faculty performance or attitude towards the comparison techniques. Another limitation is that we did not assess the students’ knowledge before and after the comparison assignment to evaluate the real effect on the students’ knowledge.

This study will help pharmacy educators and policymakers design effective strategies to improve the quality of pharmacy education. Identifying the challenges and barriers faced by pharmacy students during the application of international comparison assignments and exploring their effect on students’ future career choices need to be the focus of future research. Future research also should consider incorporating inferential statistical tests and reliability testing for the Likert scale to enhance the robustness of the findings. Additionally, including objective measures of learning, such as academic performance, would provide a more comprehensive assessment of the teaching approach. It would also be valuable to gather insights from faculty members regarding the effectiveness of the teaching method and any challenges they encountered during implementation. Such additions would offer a broader understanding of the approaches impact from both student and faculty perspectives.

## Conclusion

Using an international comparison of contrast assignments as an interactive teaching method can positively impact students’ academic and professional development. Students can gain greater knowledge of the moral and legal standards that guide pharmacy practices by contrasting various examples and circumstances. This method can also aid students in recognizing potential legal and ethical challenges that may arise in their future practice. The findings indicate that gender had a significant effect on the International Perspective and Skills domains, with females scoring higher than males. Overall, adding learning by comparison to regulation and ethics courses can help pharmacy students develop critical thinking abilities and prepare them to make wise decisions about their professions of choice.

### Summary

Learning through international comparison assignments can significantly impact students’ academic performance. This study aimed to identify students’ perceptions of using the international comparison as an interactive teaching method in learning ethical regulation and law courses. This cross-sectional study used a mixed methods approach that included a survey and open-ended questions encouraging students to reflect about their experiences were included in the survey. This study showed that learning by using an international comparison of contrast assignments as an interactive teaching method can positively impact students’ academic and professional development by helping them develop critical thinking, research, writing, cultural awareness, and communication skills.

## Supporting information

S1 Appendix Copy of the questionnaire. (DOCX)

S2 Appendix Sample student comments. (DOCX)

S1 File Data. (XLSX)

## Abbreviations

LMS: learning management system
PBL: project-based learning
PIS: participant information statement
SA: Saudi Arabia
