# Supplementary figures and images for: The use of international comparison as interactive teaching method in pharmacy education
Source: PLoS One. 2025 Dec 11;20(12):e0338269. doi: 10.1371/journal.pone.0338269 (PMC12697984; doi:10.1371/journal.pone.0338269)

**Appendix 1**: Copy of the questionnaire
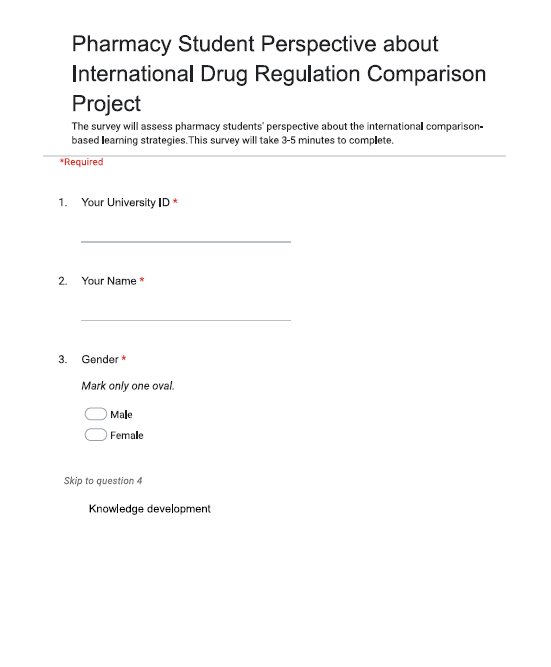


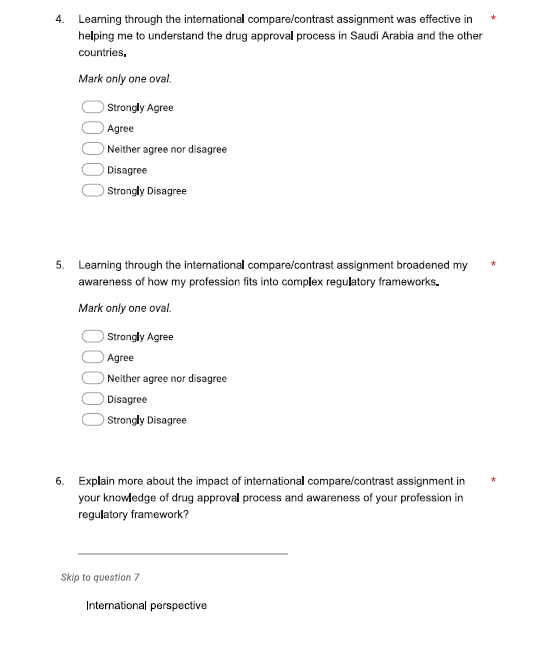

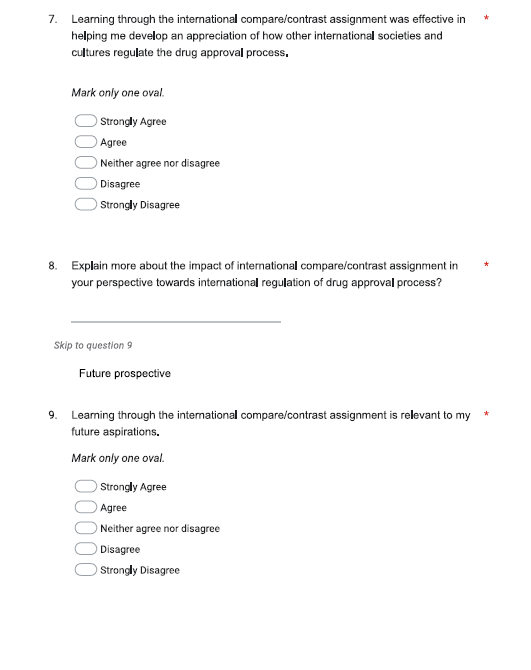

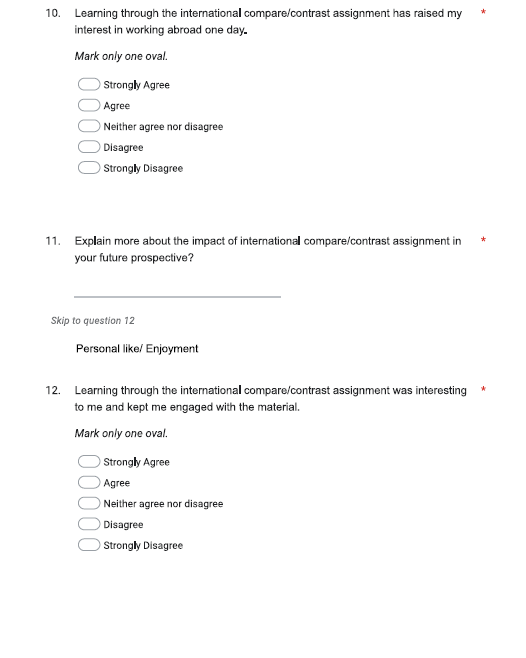

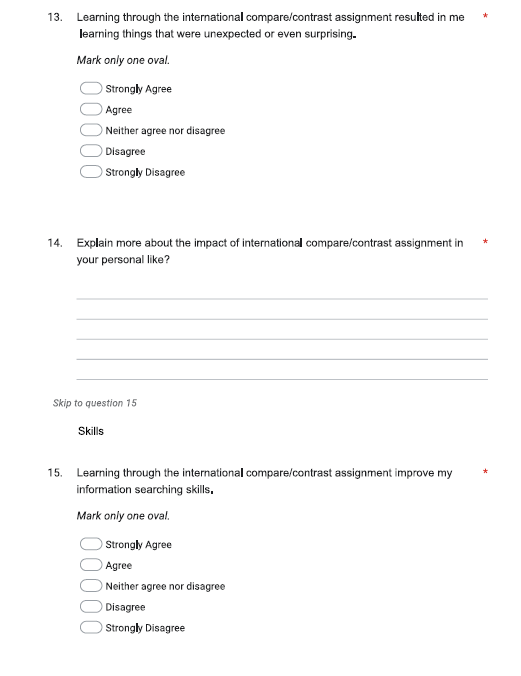

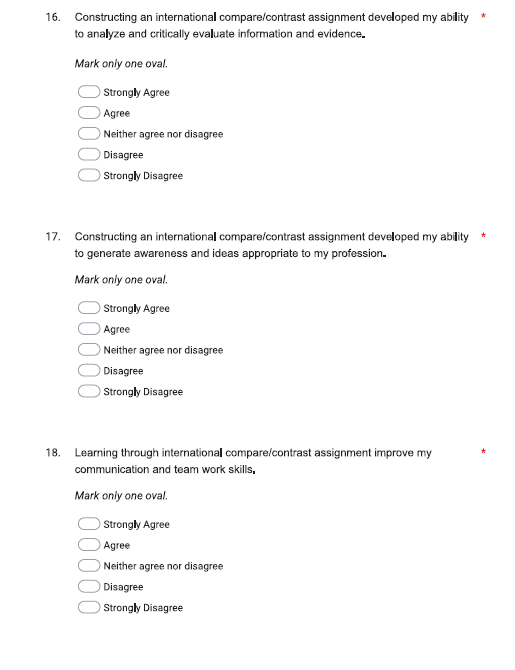


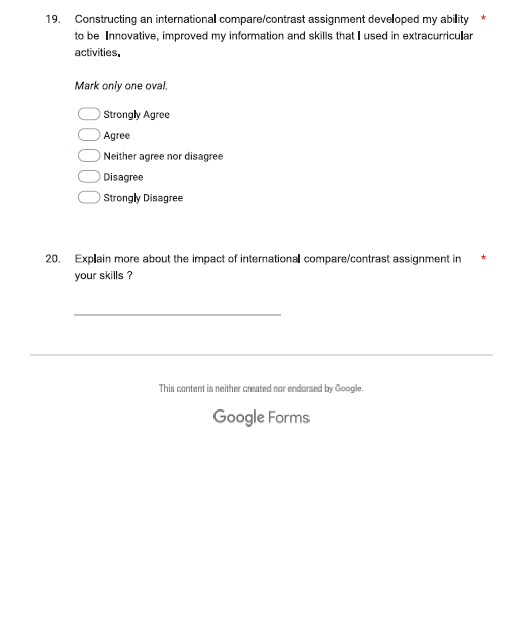

Supplement: S1 Appendix — (DOCX) [file pone.0338269.s001.docx]
