# Supplementary material for: The use of international comparison as interactive teaching method in pharmacy education
Source: PLoS One. 2025 Dec 11;20(12):e0338269. doi: 10.1371/journal.pone.0338269 (PMC12697984; doi:10.1371/journal.pone.0338269)
Supplement: S2 Appendix — (DOCX) [file pone.0338269.s002.docx]

**Appendix 2**: Sample Student Comments. S# = Student number (used to indicate individual student responses while maintaining anonymity).

| Knowledge Development   - Improves our understanding of Saudi regulatory framework for drug approval through knowing the differences between the regulations in Saudi and other countries. S161 - It's important for pharmacists to know about the approval process and compare it with that in other countries. Because we can know what the advantages and disadvantages in our drug approval process is. S47 - Since I am a pharmacy student here in Saudi Arabia, I found that this assignment has helped me a lot to understand the drug approval process that happens here and all the guidelines that it follows, and that each country has its own guidelines. S21 - Improve my pharmacist practice and skills when I know more about other countries' drug approvals; this may give me an opportunity to change some in the future. S2 - It shows me that different countries have different ways of handling different types of drugs, and the time it takes to do so varies between them, but that doesn't mean they have a fully different process for accepting that there are some similarities between the countries too. S214 - A mind opening assignment to the diversity between systems and regulatory affairs, for now I know the difference between our Saudi drug approval system and the emirates system and hopefully I will learn more. S43 - It is an eye-opening assignment with many different ideas. Students understand the drug approval procedure in both our country and the compared one, the differences in regulations and drug processes of approval, and in general with the regulatory operations. S57 - I really learned a lot doing research for the assignment, especially as a pharmacist who’s interested in the field of approval processes. But I think that we can learn even more and better utilize the information that we already have to make a presentation as groups (about the international process alone) to get to know other countries as well. S72   International Perspective   - In my opinion, due to the varying requirements for the approval of new medicines in different countries, the cost of these drugs can increase and their availability can be delayed. In developing nations, the lack of regulatory capacity can also prevent new medications from being approved. S215 - If you learn about one applicable system in your country that is used without knowing how other systems work, then you are just looking from one point of view. I guess such knowledge would make you able to detect flaws in systems and adjust them (if you are the one responsible for adjusting them) towards perfection. S43 - I learned how your rout of approving the drug depend on your goals of promoting the drug for example do you want to promote in one country on EU or more than one country. S23 - In every country there are different regulations for approving the drug, but some countries' regulations are more strict than others, so in comparison between two drugs I might prefer the one that got approval in the more strict country. S38 - Each country has procedures and steps to follow and focus on, and looking at more than one country has given me more experience in the subject and some points that are important in drug safety, and it is important to verify them before approval. S177 - The drug approval process differs from one place to another, despite having the same core values. In Saudi Arabia, and many other Arab and Muslim countries, we strongly consider a religious aspect to the drug approval process. Cultural and geographical differences will set one country apart from the others. S148 - In my group, we have chosen the Jordan FDA, which is almost identical in its drug approval process to ours. And that made me realize that by acknowledging our SFDA because it was founded before the JFDA, they adopted our regulations for drug approval, which may imply that the SFDA is considered a reference in some countries. S95   Future prospective   - Because I aspire to be a researcher in the future and own patents, it was useful to know the procedures, even if in a simple way, in different countries to expand my understanding of drug approval processes and other procedures. S182 - It’s going to have an impact on my choices of working abroad as the regulations are different for each country. S118 - Before the project, I had never thought about working for the Saudi FDA or other regulatory agencies, but after I found this very interesting, it will be one of my career choices in the future. S9 - It is giving a preconception about the role of pharmacist in drug regulation process and what is the real responsibilities that I will deal with if I become part of the process. S11 - In my feature-length perspective, I think we can see the pros and cons in our country and reduce the cons through getting experience from other regulations. S68 - The regulatory framework and drug approval process are both quite complicated. This assignment showed me how tough and complex systems are in general, which makes it challenging if we choose to work abroad because we will need to know all of the points in each country independently. S70 - My interest in my future profession comes first from direct contact with the patients or students in my country, and by saying that, I don't think that regulatory affairs or working abroad would be in my interest from my present or future perspective. At least, that is my opinion at this moment after this assignment. S95 - Actually, I am not considering working there in the future, but I believe it’s important for each pharmacist to have a background in drug approval around the world. S152   Personal Enjoyment   - It was an interesting assignment, and I was excited to know more about the regulatory process framework around the world. Since we don’t get to present this assignment, I had to ask my colleagues to give a brief summary of their findings for their assigned country. S12 - It’s interesting how each country has its own method, which makes me more fascinated by learning about the regulations of each country. S23 - I enjoyed reading the process of both countries but had problems with understanding the process of the country (Australia) due to the differences in terminology. S93 - Our project was a comparison between KSA and the US, which is familiar because we passed through it during courses. I wish it was a different country that we didn’t know much about, like Germany or Japan, that would be more interesting. S100 - I liked how I got to learn something new, as I never imagined I would know the drug approval process of another country extremely well. S103 - I always wondered if the drug approval process were the same in all countries, and my international compare and contrast assignment helped me read more about this. And answer my question. S16   Assessment skills:   - Certainly, many skills have developed, such as analysis, a passion for reading and comparison, and creative thinking. S47 - It developed my communication and searching skills by reducing conflicts between related concepts and improving comprehension. S52 - I got to develop my searching skills significantly for example, I encountered several resources and different documents that seems identical at first, then after reading them more carefully it turned out to be an updated version of each other. S72 - This assignment requires several skills to have or even to take the chance and develop them, and they include writing skills, communication skills, cooperation skills, respect for other perspectives and opinions, unity in determining the final document to be submitted, and time management. S95 - It improved my reading, summarizing, and paraphrasing skills. Alongside also improving my teamwork skills. S130 - This assignment was another necessary step in our progress toward graduating with excellent research and critical thinking skills. S148 |
| --- |
